# Effects of parecoxib on analgesia benefit and blood loss following open prostatectomy: a multicentre randomized trial

**DOI:** 10.1186/s12871-015-0015-y

**Published:** 2015-03-09

**Authors:** Daniel Dirkmann, Harald Groeben, Hassan Farhan, David L Stahl, Matthias Eikermann

**Affiliations:** 1 Klinik für Anästhesiologie und Intensivmedizin, Universität Duisburg-Essen, Universitätsklinikum Essen, Hufelandstrasse 55, Essen, D-45144 Germany; 2 Klinik für Anästhesiologie, Intensivmedizin und Schmerztherapie, Kliniken Essen Mitte, Henricistrasse 92, Essen, 45136 Germany; 3 Department of Anesthesia, Critical Care and Pain Medicine, Massachusetts General Hospital, 55 Fruit Street, Boston, MA 02114 USA

**Keywords:** Analgesics non-opioids, Parecoxib, Analgesics opioids, Morphine, Pain, Postoperative pain

## Abstract

**Background:**

This multi-centre, prospective, randomized, double-blind, placebo-controlled study was designed to test the hypotheses that parecoxib improves patients’ postoperative analgesia without increasing surgical blood loss following radical open prostatectomy.

**Methods:**

105 patients (64 ± 7 years old) were randomized to receive either parecoxib or placebo with concurrent morphine patient controlled analgesia. Cumulative opioid consumption (primary objective) and the overall benefit of analgesia score (OBAS), the modified brief pain inventory short form (m-BPI-sf), the opioid-related symptom distress scale (OR-SDS), and perioperative blood loss (secondary objectives) were assessed.

**Results:**

In each group 48 patients received the study medication for 48 hours postoperatively. Parecoxib significantly reduced cumulative opioid consumption by 24% (43 ± 24.1 mg versus 57 ± 28 mg, mean ± SD, p=0.02), translating into improved benefit of analgesia (OBAS: 2(0/4) versus 3(1/5.25), p=0.01), pain severity (m-BPI-sf: 1(1/2) versus 2(2/3), p < 0.01) and pain interference (m-BPI-sf: 1(0/1) versus 1(1/3), p=0.001), as well as reduced opioid-related side effects (OR-SDS score: 0.3(0.075/0.51) versus 0.4(0.2/0.83), p=0.03). Blood loss was significantly higher at 24 hours following surgery in the parecoxib group (4.3 g⋅dL^−1^ (3.6/4.9) versus (3.2 g⋅dL^−1^ (2.4/4.95), p=0.02).

**Conclusions:**

Following major abdominal surgery, parecoxib significantly improves patients’ perceived analgesia. Parecoxib may however increase perioperative blood loss. Further trials are needed to evaluate the effects of selective cyclooxygenase-2 inhibitors on blood loss.

**Trial registration:**

ClinicalTrials.gov Identifier: NCT00346268

## Background

Ineffective postoperative pain control still remains an unsolved problem [1], resulting in prolonged hospital stay and increased hospital costs [2]. Individualized, procedure specific postoperative analgesia has been advocated in order to solve this issue [3]. However, despite the growing evidence that there are procedure specific differences in postoperative pain, guidelines are generalized for most surgical procedures [3] and opioids are being used as the mainstay of analgesia [4]. However, their use is associated with well known side effects, which may affect patient satisfaction, length of hospital stay, and increase cost of care [5,6]. Non-steroidal anti-inflammatory drugs (NSAIDs), decrease perioperative opioid consumption and opioid-related side effects [7]. However, their use is associated with adverse events such as surgical bleeding and ulcer formation [7]. Selective cyclooxygenase-2 (COX-2) inhibitors have been proposed to be a safe alternative for several years. Parecoxib (a prodrug of valdecoxib) is a parenterally selective cyclooxygenase-2 (COX-2) inhibitor that reduces postoperative opioid consumption, following thyroid surgery [8], hernia repair [9], gynecological laparotomy [10], total hip [11] and knee arthroplasty [12], as well as spine surgery [13]. However, the use of parecoxib in cardiac surgery has been associated with an increased incidence of cardiovascular events in at-risk patients [14,15]. Furthermore, long-term use of two other COX-2 inhibitors (namely celecoxib and rofecoxib) has been associated with an increased cardiovascular risk in large trials that sought to assess the preventive effects of these drugs on colorectal adenoma development [16,17]. In addition, there are data suggesting that COX-2 inhibitors may increase perioperative blood loss in non cardiac surgery [11].

The discovery of fraud in the publications by Reuben et al. and the subsequent retraction of 21 peer-reviewed articles on perioperative analgesia, raised additional severe concerns about what is still known about risks and benefits of COX-2 inhibitor treatment [18] and the validity of review articles including such fraudulent data has been questioned [19]. Accordingly, it seems important to gather additional perioperative data to better define the risk-benefit ratio of COX-2 inhibitor treatment in a procedure-specific fashion.

This multi-centre, prospective, randomized, double-blind, placebo-controlled study was designed to test the hypotheses that parecoxib improves patients’ postoperative analgesia without increasing surgical blood loss following radical open prostatectomy. The reduction of morphine consumption during the first 48 postoperative hours was defined as the primary objective. Patients’ benefit of analgesia, reduced pain severity and pain interference, as well as reduced opioid related side effects, as assessed by respective validated scores, were chosen as secondary objecitves. Furthermore, the effects of parecoxib on blood loss, as measured by the peri-and postoperative decrease in the serum hemoglobin concentration were defined as a secondary objective, given the conflicting evidence in the literature on this side-effect of COX-2 inhibitors.

## Methods

This investigator initiated, multi-centric, prospective, double-blind, placebo-controlled study was conducted in compliance with the ‘Declaration of Helsinki’ and according to ‘Good Clinical Practice’ guidelines, at three German hospitals. Patients were recruited between December 2006 and September 2010, when the study was prematurely terminated due to slow recruitment as a result of increasing use of robot assisted surgery at all investigational centers. The study was funded by Pfizer, Germany. Pfizer was involved in the generation of the study design. However, Pfizer did not influence the interpretation and the discussion of results. The study was registered at www.clinicaltrials.gov (Identifier: NCT00346268). The study was reviewed and approved by the ethics committee of the University Duisburg-Essen (Ethik-Komission der Universität Duisburg Essen, Robert-Koch-Straße 9–11, 45147 Essen, Germany, protocol number: A3481066, date of approval 25.09.2006). All patients gave written informed consent.

### Inclusion and exclusion criteria

We included patients scheduled for elective radical open prostatectomy with an American Society of Anesthesiologists (ASA) physical status of I or II, who did not have a high risk of developing an acute coronary event within the next 10 years, according to the Prospective Cardiovascular Münster Heart Study (PROCAM) [20].

To help improve the validity of our results whilst maximizing patient safety, patients with congestive heart failure or established ischemic heart disease, peripheral and/or cerebrovascular arterial disease, or those with a history of coronary artery bypass graft (CABG) procedure were excluded. Additional exclusion criteria were a history of asthma or bronchospasm that required treatment with oral glucocorticoids, inflammatory bowel disease, chronic or acute renal or hepatic disease, coagulopathy, and adverse events after previously taking acetylsalicylic acid NSAIDs. We did not include patients with active or suspected gastrointestinal ulceration or bleeding, or a history of alcohol, analgesic, or narcotic abuse. Furthermore, individuals with known laboratory abnormality of aspartate aminotransferase (AST) or serum glutamic oxaloacetic transaminase [SGOT]) or alanine aminotransferase (ALT) or serum glutamic pyruvic transaminase [SGPT]) greater than 1.5 times the upper limit normal, or creatinine greater than 1.5 times the upper limit of normal were excluded. Finally we excluded patients on antidepressants, hypnotics, opioids, NSAIDs, antihistamines, anxiolytics, sedatives, systemic corticosteroids if the drugs were given during the 24 hours prior to surgery, except for routine preoperative anxiolytic medication. Long-acting NSAIDs (e.g., oxaprozin, piroxicam), acetylsalicylic acid, or other anti-platelet drugs were stopped 7 days before the first dose of study medication.

### Prior and concomitant medications and procedures

On the evening before surgery all subjects received dikaliumclorazepate (Tranxilium) 20 mg by mouth for sedation. On the morning prior to surgery all subjects received midazolam (Dormicum) 7.5 mg orally for anxiolysis.

All patients received general anesthesia using volatile anesthetics (desflurane or isoflurane) and fentanyl. The remaining management of general anesthesia was left to the discretion of the individual anesthesiologist. None of the patients received neuraxial analgesia. During open prostatectomy, after removal of the prostate and placement of the urinary catheter, at least one drain was placed in the perivesical space. At the end of the operation patients were extubated and transferred to the intensive or intermediate care unit for monitoring overnight. Oral fluid intake was permitted on the day of surgery. Crystalloid infusions were administered intravenously to maintain hydration during this period (up to 3,000 mL). In the evening following surgery, all subjects received enoxaparin 20 mg as thrombembolism prophylaxis. Patients also received up to 20 mg/d of dikaliumclorazepate (Tranxilium) if requested.

### Randomization and blinding

Subjects were randomized upon arrival to the post-anesthesia care unit (PACU), according to a specific identification that had been assigned at the preoperative visit. The clinical site’s pharmacist or authorized site personnel allocated the subject into either the parecoxib or the placebo arm using a computer generated random list. In order to preserve the double-blind assignment, treatments were prepared by a third person not being involved in the evaluation of subjects. All study medications were administered as a clear solution using 2 ml syringes.

### Study medication and rescue analgesia

The first dose of the study medication (parecoxib 40 mg or placebo intravenously) was administered upon patients’ arrival in the PACU by the anesthesiologist. Subsequent doses of the study medication (parecoxib 20 mg or placebo) were administered by a study staff nurse every 12 hours (±1 h) until postoperative day 2 (48 ± 1 h) after skin closure. Patients received patient controlled analgesia using morphine (1 mg/ml) for postoperative analgesia with the following setting: no continuous infusion, bolus-dose 1 mg, lock-out time 10 min, 4-h dose limit 40 mg).

### Measurements

All subjects underwent scheduled visits 24 (±1 h) and 48 (±1 h) hours, after receiving the first dose of the study medication. Opioid consumption and all items needed to calculate the modified-brief pain inventory-short form (m-BPI-sf) (pain perception (pp) score and pain interference (pi) score), the opioid-related symptom distress scale (OR-SDS), and the Overall Benefit of Analgesic Score (OBAS), were assessed.

The m-BPI-sf consists of a four-item pain severity domain (worst pain, least pain, average pain, and current pain) and a seven-item pain interference domain (general activity, mood, walking ability, relations with others, sleeping, coughing, deep breathing, and concentration). Both domains are rated on a 10 point scale, and the two domains are reported as composite scores calculated as the average of the respective items [21]. The OR-SDS assesses 3 symptom distress dimensions (frequency, severity, bothersomeness) for 12 opioid related symptoms on a 4 point scale. The average of these latter dimensions reflects the respective symptom specific score. The composite OR-SDS is calculated as the average of the 12 symptom specific scores. The OR-SDS is a validated tool for assessing if reduction in opioid use translates into a reduction of the incidence and severity of opioid related side effects [22]. In order to overcome the limitations of every single score, we developed and validated the OBAS by using pain scores, opioid consumption, as well as the m-BPI-sf, and OR-SDS, previously [23]. The OBAS combines measurements of pain intensity, opioid related adverse events, and also patients’ satisfaction (global evaluation). Accordingly, we believe it is an important outcome variable that reflects a patient’s subjective benefit from postoperative multimodal pain therapy.

Furthermore, we measured the intra-and postoperative decrease in serum hemoglobin concentration (Hb), and transfusion requirements intraoperatively and during 48 hours following skin closure [24]. More specifically, intra operative blood loss was defined as: [Hb g⋅dL^−1^]pre–[Hb g⋅dL^−1^]post + intraOP RBCU; where ([Hb g⋅dL^−1^]pre is the blood hemoglobin concentration preoperatively, [Hb g/dL]post is the blood hemoglobin concentration assessed postoperatively, and intraOP RBCU is the number of red blood cell units (RBCU) transfused during prostatectomy. Furthermore, we calculated the total blood loss as: [Hb g/dL]pre–[Hb g/dL]@48 + RBCU during 48 hours; where ([Hb g/dL]pre is the blood hemoglobin concentration preoperatively, [Hb g⋅dL^−1^] @48 is the blood hemoglobin concentration 48 h after skin closure, and RBCU during 48 hours is the number of red blood cell units (RBCU) transfused after prostatectomy during the 48 hours after skin closure. Total blood loss at 24 postoperative hours was calculated similarly. However, as there were no data available for RBCU transfused during the first 24 hours, calculations did not account for transfusions.

Serious adverse events were monitored by daily chart review, and by interviewing the Urologist in charge of this study, in order to monitor for perioperative myocardial infarction, stroke, pulmonary embolism, deep vein thrombosis, and gastrointestinal or surgical bleeding.

### Statistical analysis

A prioiri sample size calculation was performed using a two sided *t*-test with a type I error of 5%. Considering an estimated drop-out rate of 15% and an expected reduction in morphine consumption by 25%, a total of 76 individuals per group was calculated to achieve a power of 80%.

All data were analyzed using Prism 5 for Mac OS X, Version 5.0d (Graph Pad Inc., La Jolla, California). Data were tested for normality using the Kolmogorov-Smirnov test with Dallal and Wilkonson approximation of the Lillifores method, where appropriate. Data were analyzed using *t*-test, Mann–Whitney-*U* test, or chi-squared test as noted below. If not stated otherwise, data are shown as median (25th/75th percentile).

Morphine consumption (primary), and blood-loss (secondary) during the first 48 h, and variables on OBAS, m-BPI-sf score, and OR-SDS taken at 48 h after skin closure were compared between groups using Mann–Whitney-*U*-test.

We applied a multiple regression analysis model using postoperative decrease in hemoglobin concentration as the dependent variable and included independent variables that we considered might affect postoperative blood loss during parecoxib therapy: age, activated partial thromboplastin time (aPTT), Quick, platelet count, test drug.

The incidence of adverse events was compared between groups using chi-squared tests.

## Results

### Patients

**Figure 1:**
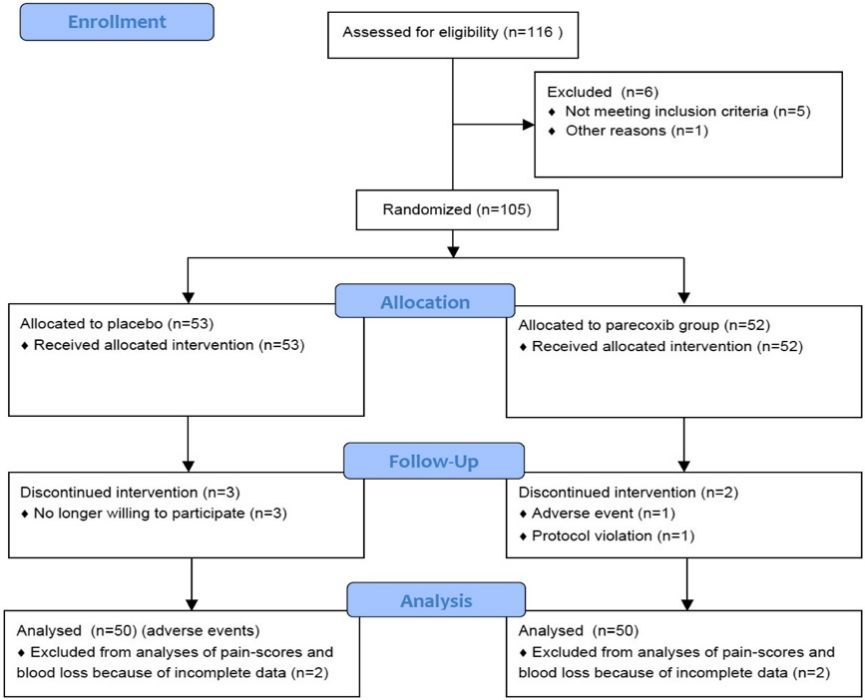
**Patient flow chart.**

A total of 105 patients (52 parecoxib, 53 placebo) were enrolled in this trial and received treatment. Of these subjects, 96 patients (48 parecoxib, 48 placebo) received the study medication for 48 hours postoperatively and had complete data sets available. One patient had to be excluded for protocol violation, another due to an adverse event (hyperhydrosis, parecoxib group), and three patients had to be excluded because of withdrawal of consent (placebo group). Two patients from each group had to be excluded since relevant data were missing (Figure 1). Of the patients included in the final analyses, 34 (17 receiving parecoxib) were recruited at investigational center one, 60 patients (31 receiving parecoxib) at investigational center two, and two patients (both receiving placebo) were recruited at investigational center three. The physical characteristics and laboratory variables were comparable in both groups (Table 1).

**Table 1:** **Descriptive data**

	Parecoxib	Placebo
Number of subjects	52	53
Subjects excluded	4	5
Age (years)	64.4 (±7.5)	65.0 (±7.2)
Age (years) range	47-83	46-75
Haemoglobin (g⋅dL^−1^)	14.5 (±1.2)	14.5 (±1.5)
aPTT (s.)	30 (±3.3)	30.1 (±3.6)
Quick (%)	100 (88 / 100)	97.5 (94 / 100)
Platelet count (x10^9^/L)	244 (±62)	224 (±56)

Data are given as numbers, mean (± standard deviation), or median (25th/75th percentile), as appropriate. There were no significant differences between groups.

### Efficacy

**Figure 2:**
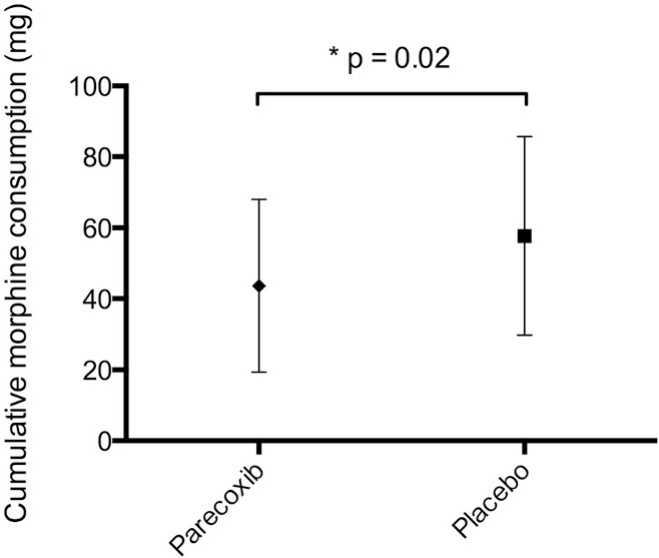
**Cumulative amount of morphine used at 48 hours following skin closure.** Mean (symbols) and standard deviation (error bars). Morphine consumption was significantly (24%) less in the parecoxib group vs. placebo.

**Figure 3:**
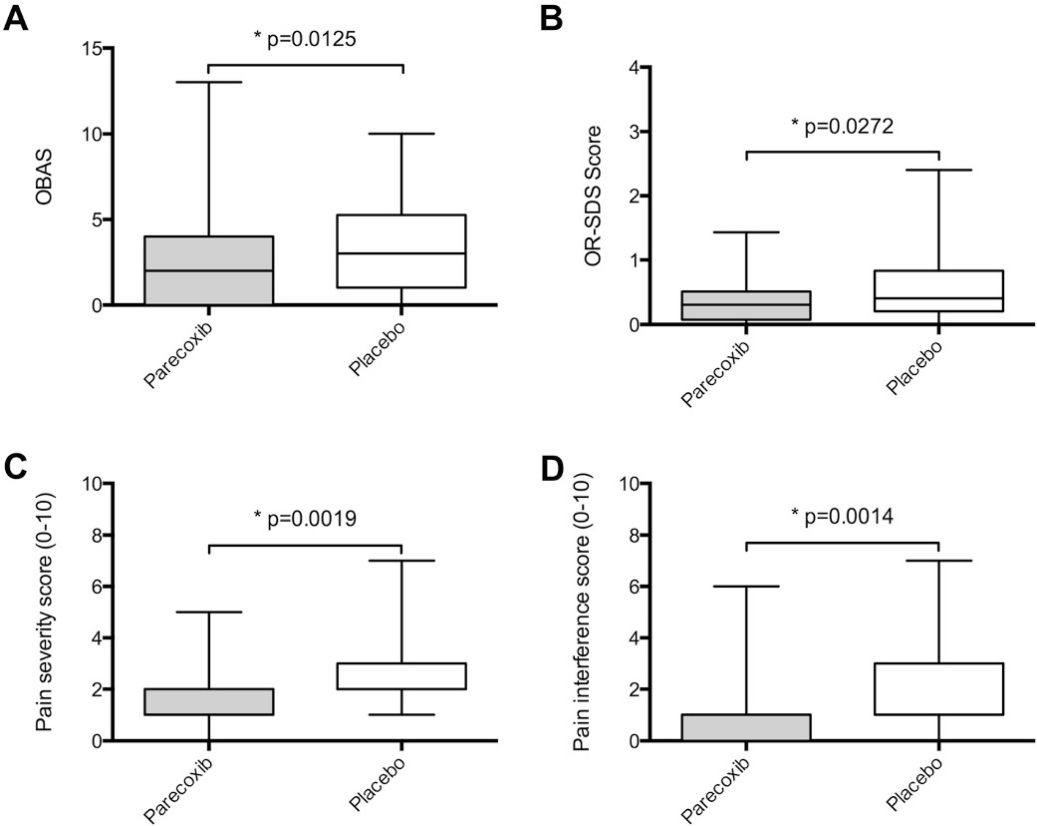
**Scoring system variables of analgesic efficacy at 48 hours following skin closure.** OBAS **(A)**, OR-SDS score **(B)**, m-BPI-sf pain perception score **(C)**, and m-BPI-sf pain interference score **(D)**. Box-plots of quartiles (boxes), median (line within box), minimum, and maximum (error bars). All measurements of analgesic efficacy were significantly less in the parecoxib group vs. placebo.

Mean morphine consumption was lower (24.4%) during the first 48 hours following surgery in subjects receiving parecoxib (43.1 ± 24.1 mg) (mean ± SD) as compared to those receiving placebo (57.1 ± 28 mg, p=0.02) (Figure 2). Parecoxib administration resulted in a significantly decreased OBAS at 48 hours after the first administration (2 (0/4)) as compared to the placebo group (3 (1/5.25)) (p=0.01) (Figure 3A). Values of Opioid Related-Symptom Distress Scale (OR-SDS) were lower in patients receiving parecoxib (0.3 (0.08/0.51)) compared to the placebo group (0.4 (0.2/0.83)) (p=0.03, Figure 3B).

Calculation of the pain severity (ps) and the pain interference (pi) scores of the Modified-Brief Pain Inventory-Short Form (m-BPI-sf) revealed that parecoxib was effective in reducing patients’ pain severity (1(1/2) versus 2(2/3), p < 0.01, Figure 3C) as well as pain interference with patients’ life (1(0/1) versus 1(1/3), p < 0.01, Figure 3D).

### Blood loss and transfusion requirements

Intraoperative decrease in Hb was not significantly different between both groups (p=0.26). Median decrease in Hb was 3.2 g⋅dL^−1^(2.1/3.7) in the parecoxib group and 2.3 g⋅dL^−1^(1.7/3.8) in the placebo group.

**Figure 4:**
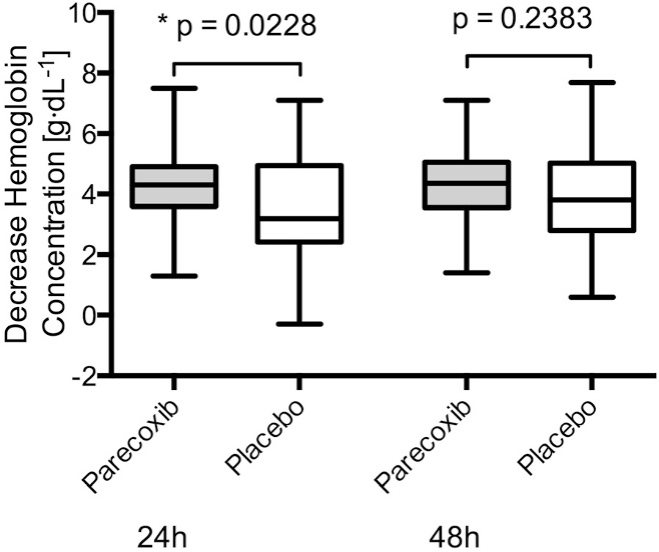
**Decrease in hemoglobin concentration (Hb) measured as: Hb [g⋅dL**^**−1**^**] preoperatively–Hb [g⋅dL**^**−1**^**] at 48 hours following skin closure + RBCU during 48 hours; where RBCU during 48 hours is the number of red blood cell units (RBCU) transfused after open prostatectomy until 48 h after skin closure.** Box-plots of quartiles (boxes), median (line within box), minimum, and maximum (error bars). Decrease in Hb was significantly higher in the parecoxib group vs. placebo at 24 hours.

Decrease in Hb during the first 24 hours following skin closure was however significantly greater in the parecoxib group (4.3 g⋅dL^−1^(3.6/4.9)) as compared to the placebo group (3.2 g⋅dL^−1^(2.4/5.0) (p=0.02) (Figure 4). Multiple regression analysis using decrease in Hb assessed at 24 hours postoperatively as the dependent variable and age, activated partial thromboplastin time (aPTT), Quick, platelet count, and test drug as independent variables confirmed that the test drug (i.e. parecoxib or placebo) was the only independent factor associated with higher decrease in Hb during 24 hours, postoperatively (p=0.026). Data on the variables included are listed in Table 1. However, the effect was no longer statistically significant when analyzing the decrease in Hb at 48 hours. The median decrease in Hb at 48 hours was 4.4 g⋅dL^−1^(3.8/5.4) in the parecoxib group and 3.85 g⋅dL^−1^(2.8/5.4) in subjects receiving placebo (p=0.12, Figure 4).

Transfusion requirements were similar between groups. One patient from each group had 1 RBCU transfused within the first 48 hours following skin closure. Four subjects from the parecoxib group and 1 subject from the placebo group had 2 RBCU transfused within the first 48 hours after surgery.

### Adverse events

All patients were included in the analyses of safety. Throughout the study period, a total of 116 adverse events (AEs) were observed in 43 subjects (83%) in the parecoxib group and 109 AEs in 42 subjects (79%) in the placebo group, respectively. The most common adverse events, occurring in 2 or more subjects are listed in Table 2. There were no statistical differences in the frequencies of the listed adverse events between the treatment and the placebo group, respectively, as determined using a Fishers exact test. Most adverse events were classified mild (n=156) to moderate (n=62) in both groups. However, serious or severe adverse events (n=7) were reported in two subjects in the parecoxib group (thrombocytosis and hyperhidrosis), and 5 subjects in the placebo group (diarrhea, pyrexia, confusional state, and hemorrhage). Only one patient in the parecoxib group discontinued treatment, secondary to severe hyperhidrosis, which started 20 minutes after receiving the study drug. Of the adverse events in the parecoxib arm, a total of three (hypokalemia, acute renal failure, and hyperhidrosis) were classified as being treatment related.

**Table 2:** **Incidence of adverse events**

	Parecoxib (n=52)	Placebo (n=53)	p-value (Parecoxib vs. Placebo)
Anemia	6 (12)	4 (8)	0.53
Vertigo	0 (0)	3 (6)	0.24
Abdominal pain	3 (6)	1 (2)	0.36
Constipation	2 (4)	7 (13)	0.16
Diarrhea	4 (8)	8 (15)	0.36
Flatulence	6 (12)	10 (19)	0.42
Nausea	12 (23)	16 (30)	0.51
Vomiting	9 (17)	4 (8)	0.15
Pyrexia	1 (2)	4 (8)	0.36
Urinary tract infection	8 (15)	7 (13)	0.79
Wound infection	2 (4)	3 (6)	1.0
Back pain	2 (4)	0 (0)	0.24
Hypokaliemia	2 (4)	2 (4)	1.0
Dizziness	2 (4)	2 (4)	1.0
Paresthesia	2 (4)	2 (4)	0.24
Confusion	0 (0)	2 (4)	0.5
Insomnia	3 (6)	1 (2)	0.36
Bladder pain	0 (0)	2 (4)	0.5
Urinary retention	2 (4)	1 (2)	0.62
Cough	1 (2)	2 (4)	1.0
Oropharyngeal pain	3 (6)	2 (4)	0.68
Deep vein thrombosis	2 (4)	0 (0)	0.24
Hypertension	6 (12)	4 (8)	0.53
Hypotension	2 (4)	0 (0)	0.24
Lymphocele	6 (12)	2 (4)	0.16

There were no differences in the incidence of adverse events between parecoxib and placebo. Results are given as numbers (%) of patients reporting an adverse event.

## Discussion

This study demonstrates that postoperative parecoxib co-administration with morphine in patients undergoing radical open prostatectomy significantly decreases morphine consumption by an average of 24%. The finding that parecoxib reduces opioid consumption as part of a multimodal postoperative analgesic strategy is not unanticipated, as this effect has been previously demonstrated. However, this study improves upon previous studies by assessing more than just opioid consumption. We assessed the effect of multimodal analgesia with parecoxib on patient satisfaction and side effects using previously validated clinically relevant scoring systems, finding that multimodal analgesia improves the quality of pain relief whilst also reducing unpleasant side effects. However, our study also suggests that parecoxib use in patients undergoing radical open prostatectomy may be associated with increased perioperative blood loss in the first 24 hours after surgery.

### Postoperative analgesia

In 2001, the Joint Commission on Accreditation of Hospital Organizations advocated pain management a priority in delivering good care [25]. This led to an increase in the prescription of opioids, especially in surgical patients, in a bid to minimize patient pain levels. Opioids are highly effective analgesics in the treatment of postoperative pain, however their use is associated with potentially serious and often distressing side effects for patients. Respiratory depression, alterations in mental status, nausea, vomiting and constipation/ileus are all known side effects of opioid medication [26]. Multimodal analgesia strategies aim to reduce opioid consumption and thus opioid side effects whilst also improving overall analgesic management and patient satisfaction by combining opioid and non-opioid medications in the treatment of postoperative pain [6]. NSAIDs are commonly used in these multimodal strategies and have been proven to reduce postoperative opioid consumption [7].

Non-selective NSAIDs are not suitable for the management of pain in the immediate postoperative period as they inhibit COX-1 expressed in platelets, leading to reduced platelet aggregation and an increased risk of bleeding. Selective COX-2 inhibitors avoid this unwanted side effect. The advent of Parecoxib, a selective COX-2 inhibitor that can be administered parenterally, made the use of NSAIDs as part of a multimodal pain therapy regime in surgical patients more feasible. Parecoxib has been licensed for use in Europe for the short-term treatment of postoperative pain. The efficacy of this drug in reducing opioid consumption and side effects following major abdominal surgery has not been extensively investigated.

### Efficacy in reducing opioid consumption

The efficacy of analgesic drugs for postoperative analgesia is considered to be procedure specific [27], and most studies focus on the reduction of opioid requirements as their primary outcome measure [7]. In our present study, parecoxib analgesia resulted in an average reduction of cumulative morphine requirements by 24% compared to placebo in patients undergoing open prostatectomy. This finding is in accordance with previous studies describing an opioid sparing effect of COX-2 inhibitors in the postoperative period [8-13]. However, opioid sparing is not by itself considered a clinically meaningful endpoint [7,28]. Thus, we examined other measurements assessing patients’ benefits from multimodal analgesia.

### Parecoxib and patient satisfaction

Most studies assess the incidence of adverse events but seldom use well established validated scores, like the OR-SDS or the pain interference score derived from the m-BPI-sf [29]. Since both pain related symptoms and opioid related side effects are associated with patient satisfaction [30], we performed additional assessment of the OBAS which we have recently developed and validated. As demonstrated, the OBAS correlated much better with patient satisfaction than analyzing pain scores alone. Furthermore, the OBAS yields higher resolution of analgesic treatment effects of COX-2 inhibitors than pain scores, OR-SDS and m-BPI-sf [23]. In the present study, the opioid sparing effect of postoperative parecoxib translated into a significant reduction of OBAS, OR-SDS, and m-BPI-sf scores, indicating that patients perceived significant benefit from parecoxib, namely reduced pain intensity, reduced opioid associated side effects, and less interference with their lives (general activity, mood, walking ability, relations with others, sleeping, coughing, deep breathing, and concentration) [21]. These findings are in accordance with other studies assessing OR-SDS and/or m-BPI scores [13,31,32] and indicate that opioid sparing effects of non-opioid analgesics may translate into clinical benefit in patients analgesia.

### Parecoxib and postoperative bleeding

In our study, the postoperative use of parecoxib was not associated with increased bleeding, as measured by the total blood loss at 48 hours postoperatively, a priori defined secondary outcome. However, there was a trend towards higher blood loss at this predefined time interval and a trend towards higher transfusion requirements (5 patients in the parecoxib group vs. 2 patients in the placebo group receiving any RBCU transfusion). Furthermore, the incidence of postoperative anaemia was 1.5 times higher in patients receiving parecoxib. However, this difference was not found to be statistically significant. In addition, analyses of the postoperative decrease in Hb revealed a higher decrease in Hb in patients receiving parecoxib during the first 24 h following surgery compared to placebo. The lack of statistical significance in the total blood loss at 48 hours postoperatively might be due to the relatively small size of the study population. While use of classical NSAIDs is associated with approximately a six fold increase in surgical bleeding complications (2.4%) compared to placebo (0.4%) [33], COX-2 inhibitors are generally considered to not increase bleeding risk. However, a trend towards an increased incidence of postoperative anemia associated with parecoxib (14.1% vs. 10%) has been previously reported [11]. However, given the data by Malan et al. [11] as well as the lack of statistical significance in our data, the effect of parecoxib on postoperative bleeding is still not clearly defined, thus warranting further research in order to ensure patient safety in the postoperative period.

As stated earlier, COX-2-inhibitors do not affect platelet aggregation, as shown by impedance aggregometry, thromboelastometry, and platelet function analyzer (PFA-100) assays [34,35]. Nevertheless, COX-2 inhibitors may increase blood loss via drug interactions [36,37]. Parecoxib and its metabolite valdecoxib are suspected to potentiate warfarin’s effects since both COX-2 inhibitors exert inhibitory effects on CYP2C9 [38], an enzyme that also metabolizes warfarin. Co-medication with warfarin and parecoxib has been shown to increase the propensity to bleeding [38]. In addition, we speculate that it might be possible that COX-2 inhibitors, similar to aspirin [39] may affect fibrinolysis and that increased blood loss might be procedure-specific [40].

### Limitations

Our study has certain limitations. Most centers now prefer laporascopic or robot-assisted-laporascopic versus open prostatectomy and open radical postatectomy is only performed in about 25% of cases [41]. Accordingly, one could argue that our study population does not represent a typical cohort of patients undergoing prostatectomy. However, pain and morphine consumption following open radical or robot-assisted laparoscopic prostatectomy are similar [42]. Another limitation of our study relates to unbalanced patient enrollment in the three centers. While investigational centers one and two enrolled 34 and 60 patients, respectively, center three enrolled only 2 patients. Removing these two patients from our analyses did not change our results.

Finally, our data regarding blood loss have to be interpreted cautiously. First of all our study was designed to assess blood loss at 48 hours not as a primary, but as a secondary outcome measure and the study was originally powered based on the expected opioid sparing effect. Perioperative blood loss is affected by several confounding factors such as surgical technique. Our finding of increased blood-loss in the parecoxib group is hypothesis generating. Since our findings are in line with the observation of others [11] we feel that the effects of COX-2 inhibitors on blood loss should be further evaluated in future trials.

## Conclusions

In summary, our data show that the perioperative use of parecoxib for adjunctive analgesia following open prostatectomy is associated with significant opioid sparing that translates into clinical analgesic benefit to patients. Parecoxib may however increase perioperative blood loss.

## References

[1] Fletcher D, Fermanian C, Mardaye A, Aegerter P (2008). A patient-based national survey on postoperative pain management in France reveals significant achievements and persistent challenges. Pain.

[2] Morrison RS, Magaziner J, McLaughlin MA, Orosz G, Silberzweig SB, Koval KJ (2003). The impact of post-operative pain on outcomes following hip fracture. Pain.

[3] Gerbershagen HJ, Aduckathil S, van Wijck AJM, Peelen LM, Kalkman CJ, Meissner W (2013). Pain intensity on the first day after surgery: a prospective cohort study comparing 179 surgical procedures. Anesthesiology.

[4] Power I (2011). An update on analgesics. Br J Anaesth.

[5] Philip BK, Reese PR, Burch SP (2002). The economic impact of opioids on postoperative pain management. J Clin Anesth.

[6] White PF (2005). The changing role of non-opioid analgesic techniques in the management of postoperative pain. Anesth Analg.

[7] Marret E, Kurdi O, Zufferey P, Bonnet F (2005). Effects of nonsteroidal antiinflammatory drugs on patient-controlled analgesia morphine side effects: meta-analysis of randomized controlled trials. Anesthesiology.

[8] Gehling M, Arndt C, Eberhart LHJ, Koch T, Krüger T, Wulf H (2010). Postoperative analgesia with parecoxib, acetaminophen, and the combination of both: a randomized, double-blind, placebo-controlled trial in patients undergoing thyroid surgery. Br J Anaesth.

[9] Kyriakidis AV, Perysinakis I, Alexandris I, Athanasiou K, Papadopoulos C, Mpesikos I (2010). Parecoxib sodium in the treatment of postoperative pain after Lichtenstein tension-free mesh inguinal hernia repair. Hernia.

[10] Barton SF, Langeland FF, Snabes MC, LeComte D, Kuss ME, Dhadda SS (2002). Efficacy and safety of intravenous parecoxib sodium in relieving acute postoperative pain following gynecologic laparotomy surgery. Anesthesiology.

[11] Malan TP, Marsh G, Hakki SI, Grossman E, Traylor L, Hubbard RC (2003). Parecoxib sodium, a parenteral cyclooxygenase 2 selective inhibitor, improves morphine analgesia and is opioid-sparing following total hip arthroplasty. Anesthesiology.

[12] Hubbard RC, Naumann TM, Traylor L, Dhadda S (2003). Parecoxib sodium has opioid-sparing effects in patients undergoing total knee arthroplasty under spinal anaesthesia. Br J Anaesth.

[13] Riest G, Peters J, Weiss M, Dreyer S, Klassen PD, Stegen B (2007). Preventive effects of perioperative parecoxib on post-discectomy pain. Br J Anaesth.

[14] Ott E, Nussmeier NA, Duke PC, Feneck RO, Alston RP, Snabes MC (2003). Efficacy and safety of the cyclooxygenase 2 inhibitors parecoxib and valdecoxib in patients undergoing coronary artery bypass surgery. J Thorac Cardiovasc Surg.

[15] Nussmeier NA, Whelton AA, Brown MT, Langford RM, Hoeft A, Parlow JL (2005). Complications of the COX-2 inhibitors parecoxib and valdecoxib after cardiac surgery. N Engl J Med.

[16] Solomon SD, McMurray JJV, Pfeffer MA, Wittes J, Fowler R, Finn P (2005). Cardiovascular risk associated with celecoxib in a clinical trial for colorectal adenoma prevention. N Engl J Med.

[17] Bresalier RS, Sandler RS, Quan H, Bolognese JA, Oxenius B, Horgan K (2005). Cardiovascular events associated with rofecoxib in a colorectal adenoma chemoprevention trial. N Engl J Med.

[18] White PF, Kehlet H, Liu S (2009). Perioperative analgesia: what do we still know?. Anesth Analg.

[19] Marret E, Elia N, Dahl JB, McQuay HJ, Møiniche S, Moore RA (2009). Susceptibility to fraud in systematic reviews: lessons from the Reuben case. Anesthesiology.

[20] Assmann G, Cullen P, Schulte H (2002). Simple scoring scheme for calculating the risk of acute coronary events based on the 10-year follow-up of the prospective cardiovascular Münster (PROCAM) study. Circulation.

[21] Cleeland CS, Ryan KM (1994). Pain assessment: global use of the brief pain inventory. Ann Acad Med Singap.

[22] Apfelbaum JL, Gan TJ, Zhao S, Hanna DB, Chen C (2004). Reliability and validity of the perioperative opioid-related symptom distress scale. Anesth Analg.

[23] Lehmann N, Joshi GP, Dirkmann D, Weiss M, Gulur P, Peters J (2010). Development and longitudinal validation of the overall benefit of analgesia score: a simple multi-dimensional quality assessment instrument. Br J Anaesth.

[24] Stahl DL, Groeben H, Kroepfl D, Gautam S, Eikermann M (2012). Development and validation of a novel tool to estimate peri-operative blood loss*. Anaesthesia.

[25] Phillips DM (2000). JCAHO pain management standards are unveiled. Jt Comm Perspect JAMA.

[26] Wheeler M, Oderda GM, Ashburn MA, Lipman AG (2002). Adverse events associated with postoperative opioid analgesia: a systematic review. J Pain.

[27] Gray A (2005). Predicting postoperative analgesia outcomes: NNT league tables or procedure-specific evidence?. Br J Anaesth.

[28] Elia N, Lysakowski C, Tramèr MR (2005). Does multimodal analgesia with acetaminophen, nonsteroidal antiinflammatory drugs, or selective cyclooxygenase-2 inhibitors and patient-controlled analgesia morphine offer advantages over morphine alone? Meta-analyses of randomized trials. Anesthesiology.

[29] Rømsing J, Møiniche S, Mathiesen O, Dahl JB (2005). Reduction of opioid-related adverse events using opioid-sparing analgesia with COX-2 inhibitors lacks documentation: A systematic review. Acta Anaesthesiol Scand.

[30] Jensen MP, Mendoza T, Hanna DB, Chen C, Cleeland CS (2004). The analgesic effects that underlie patient satisfaction with treatment. Pain.

[31] Gan TJ, Joshi GP, Zhao SZ, Hanna DB, Cheung RY, Chen C (2004). Presurgical intravenous parecoxib sodium and follow-up oral valdecoxib for pain management after laparoscopic cholecystectomy surgery reduces opioid requirements and opioid-related adverse effects. Acta Anaesthesiol Scand.

[32] Langford RM, Joshi GP, Gan TJ, Mattera MS, Chen W-H, Revicki DA (2009). Reduction in opioid-related adverse events and improvement in function with parecoxib followed by valdecoxib treatment after Non-cardiac surgery. Clin Drug Investig.

[33] Maund E, McDaid C, Rice S, Wright K, Jenkins B, Woolacott N (2011). Paracetamol and selective and non-selective non-steroidal anti-inflammatory drugs for the reduction in morphine-related side-effects after major surgery: a systematic review. Br J Anaesth.

[34] Munsterhjelm E (2006). Influence on platelet aggregation of i.v. parecoxib and acetaminophen in healthy volunteers. Br J Anaesth.

[35] Scharbert G, Gebhardt K, Sow Z, Duris M, Deusch E, Kozek-Langenecker S (2007). Point-of-care platelet function tests: detection of platelet inhibition induced by nonopioid analgesic drugs. Blood Coagul Fibrinolysis.

[36] Ibrahim A, Park S, Feldman J, Karim A, Kharasch ED (2002). Effects of parecoxib, a parenteral COX-2-specific inhibitor, on the pharmacokinetics and pharmacodynamics of propofol. Anesthesiology.

[37] Halperin D, Reber G (2007). Influence of antidepressants on hemostasis. Dialogues Clin Neurosci.

[38] Cheetham TC, Levy G, Niu F, Bixler F (2009). Gastrointestinal safety of nonsteroidal antiinflammatory drugs and selective cyclooxygenase-2 inhibitors in patients on warfarin. Ann Pharmacother.

[39] Undas A, Brummel-Ziedins KE, Mann KG (2007). Antithrombotic properties of aspirin and resistance to aspirin: beyond strictly antiplatelet actions. Blood.

[40] Nielsen JD, Gram J, Holm-Nielsen A, Fabrin K, Jespersen J (1997). Post-operative blood loss after transurethral prostatectomy is dependent on in situ fibrinolysis. BJU Int.

[41] Laird A, Fowler S, Good DW, Stewart GD, Srinivasan V, Cahill D, Brewster SF, McNeill SA. Contemporary practice and technique-related outcomes for radical prostatectomy in the UK: a report of national outcomes. BJU INt 2014 [Epub ahead of print]. 10.1111/bju.12866 25046349

[42] Webster TM, Herrell SD, Chang SS, Cookson MS, Baumgartner RG, Anderson LW (2005). Robotic assisted laparoscopic radical prostatectomy versus retropubic radical prostatectomy: a prospective assessment of postoperative pain. J Urol.

